# Farnesoid X receptor activation induces antitumour activity in colorectal cancer by suppressing JAK2/STAT3 signalling via transactivation of SOCS3 gene

**DOI:** 10.1111/jcmm.16083

**Published:** 2020-11-09

**Authors:** Shan Li, Zhengshui Xu, Jing Guo, Jianbao Zheng, Xuejun Sun, Junhui Yu

**Affiliations:** ^1^ Department of Reproductive Medicine First Affiliated Hospital of Xi'an Jiaotong University Xi'an China; ^2^ Department of General Surgery First Affiliated Hospital of Xi'an Jiaotong University Xi'an China

**Keywords:** colorectal cancer, Farnesoid X receptor, JAK2/STAT3 signalling, obeticholic acid, SOCS3

## Abstract

Farnesoid X receptor (FXR, encoded by NR1H4), a bile acid‐activated nuclear receptor, is widely implicated in human tumorigenesis. The FXR agonist obeticholic acid (OCA) has preliminarily displayed tumour suppressor potential. However, the anticancer effects of this agent on colorectal cancer (CRC) remain unclear. In this study, the treatment of colon cancer cells with OCA inhibited cell proliferation and invasion in vitro, retarded tumour growth in vivo and prevented the G_0_/G_1_ to S phase transition. Moreover, the expression of active caspase‐3, p21 and E‐cadherin was up‐regulated and the expression of cyclin D1, c‐Myc, vimentin, N‐cadherin and MMP9 was down‐regulated in OCA‐treated colon cancer cells. Mechanistic studies indicated that OCA treatment suppressed the activity of JAK2/STAT3 pathway by up‐regulating SOCS3 expression. Colivelin, an agonist of JAK2/STAT3 pathway, antagonized the tumour‐suppressive effect of OCA on colon cancer cells. Dual‐luciferase reporter and quantitative chromatin immunoprecipitation (qChIP) assays further confirmed that OCA promoted SOCS3 transcription by enhancing the binding of FXR to the FXRE/IR9 of the SOCS3 promoter. In conclusion, our study demonstrates that targeting FXR and improving its function might be a promising strategy for CRC treatment.

## 1 INTRODUCTION

Colorectal cancer (CRC) is the second most common cause of cancer‐related death.^1^ Globally, approximately 1 800 000 new cases are diagnosed as CRC every year. Due to distant metastasis and relapse, most advanced‐stage CRC indicates a poor prognosis.^2^, ^3^ The 5‐year survival rate of stage I CRC in patients exceeds 90%; however, the rate of stage IV CRC in patients is slightly higher than 10%.^4^ There has recently been substantial progress in multimodality therapy for CRC. Chemotherapy is the second major treatment type for CRC after surgical treatment. However, there are many problems with chemotherapy, such as drug resistance and side effects.^5^ Thus, more effective strategies and novel targets for chemotherapy in this malignancy are urgently required.

Epidemiological studies and animal studies have suggested that prolonged exposure to faecal bile acids contributes to the occurrence of inflammatory bowel disease (IBD) and gastrointestinal cancer.^6^ Increased amounts of bile acids generate an excess of detrimental injury on colonic mucosa including ROS‐induced DNA damage,^7^ inflammation^8^ and hyperproliferation.^9^ Farnesoid X receptor (FXR, encoded by NR1H4), a bile acid‐activated nuclear receptor, regulates metabolism homeostasis of lipid, cholesterol and glucose.^10^ Mounting evidence supports a pivotal role for FXR in human tumorigenesis. FXR exerts tumour‐promoting effect in oesophageal^11^ and pancreatic carcinomas.^12^ However, some studies conclude the opposite. FXR‐deficient mice are susceptible to developing hepatocellular carcinoma (HCC).^13^ Diminished FXR correlates with late tumour stage and often predicts a poor prognosis.^14^ The deficiency of FXR promotes intestinal inflammation and colon tumorigenesis.^15^ Conversely, activation of intestinal FXR can suppress abnormal cell growth and curtail CRC progression.^16^ Thus, targeting FXR and restoring its function might be an attractive tactics for CRC treatment.

Obeticholic acid (OCA) is a novel FXR agonist, a derivative of chenodeoxycholic acid (CDCA), and shows almost 10‐fold greater potency than CDCA.^17^ Importantly, OCA has been approved by the US Food and Drug Administration (FDA) for the treatment of primary biliary cholangitis.^18^ Recent studies indicate that OCA inhibits the malignant potential of cholangiocarcinoma^19^ and HCC.^20^ However, the anticancer effect of this agent on colon cancer cells remains to be elucidated. Herein, we evaluated the impact of OCA on the aggressive phenotype of colon cancer cells and further investigated the underlying mechanisms.

## 2 MATERIALS AND METHODS

### 2.1 Cell cultures

Colon cancer cells HT‐29 and Caco‐2 (Shanghai Institute of Cell Biology, Chinese Academy of Sciences) were all routinely cultured in DMEM supplemented with 10% foetal bovine serum at 5% CO2 at 37°C. Once the cells reached 70% confluence, they were pre‐treated with colivelin (0.5 µM) or interleukin‐6 (IL‐6, 10 ng/mL) for 6 hours and then were cultured with various doses of OCA (0, 1, 2 and 4 µM) for 48 hours.

### 2.2 Lentiviral vectors and transfection

The phU6‐EGFP‐shRNA‐SOCS3 lentiviral vectors and their control vectors were constructed and prepared by GeneChem Co., Ltd. All transfections were performed according to the manufacturer's instructions.

### 2.3 Cell growth curve, CCK8, colony formation, cell cycle and apoptosis assays

Cell growth curve and CCK8 assays were performed as described previously.^21^ For colony formation assay, three hundred cells were seeded and cultured for 14 days. Colonies (≥50 cells/colony) were counted. Cell cycle distributions were evaluated by flow cytometry as previously described.^21^ For apoptosis assay, cells were labelled with Annexin V PE/7‐AAD (BD Biosciences) according to the manufacturer's protocol as previously described.^22^ Each experiment was performed in triplicate.

### 2.4 Wound‐healing assays

Cells were cultured in 6‐well plates until confluent. Then, 3 artificial vertical lines were created with pipette tips (10 µL) in each well. The wells were washed with phosphate‐buffered saline (PBS) to remove cell debris. The cells were then cultured for an additional 48 hours. The scratch lines were imaged under a microscope, and the scratch distances were measured. Each experiment was performed in triplicate.

### 2.5 Transwell assays

Cell migration and invasion were measured by using Transwell plates (Corning) with or without Matrigel (BD). In both assays, the lower chamber was filled with 600 μL of RPMI 1640 medium containing 20% FBS. For the migration assay, the cells were trypsinized and plated at 5 × 10^4^ cells per upper chamber. The cells were incubated at 37°C for 24 hours. For the invasion assay, the upper chamber filters were pre‐coated with 50 µL of Matrigel and plated at 10 × 10^4^ cells per upper chamber. The cells were incubated at 37°C for 48 hours. After incubation, non‐migratory cells on the upper surface of the Transwell inserts were removed by washing with fresh PBS. The migratory or invading cells on the underside of the membrane were fixed with 4% paraformaldehyde and stained with 1% crystal violet. The number of cells was counted in three randomly selected fields of fixed cells under an inverted microscope. Each experiment was performed in triplicate.

### 2.6 Nude mouse xenograft assay

All animal experiments were performed in accordance with the institutional guidelines and were approved by the Laboratory Animal Center of Xi'an Jiaotong University. The 5‐week‐old female BALB/c‐nude mice were purchased from Shanghai SLAC Laboratory Animal Co., Ltd. (Shanghai, China). The mice were injected with 5 × 10^6^ HT‐29 and Caco‐2 cells into the right flanks to establish xenograft tumour model. Once the palatable xenograft tumours were established, the nude mice were treated by oral gavage of OCA (10 mg/kg/d) or DMSO solution. Tumour size was monitored using callipers every 3 days, and the tumour volume was calculated according to the formula (length × width^2^ × 0.5). At the end of the experiment, the mice were killed and the xenograft tumours were isolated and weighted.

### 2.7 RNA isolation and real‐time PCR

RNA isolation, complementary DNA (cDNA) synthesized and real‐time PCR were performed as described previously.^21^ The sequences of primers were summarized in Supplementary Table S1. Each experiment was performed in triplicate.

### 2.8 Immunohistochemistry

The protocol was performed as previously described.^21^ The extent of Ki67‐ and active caspase‐3‐stained cells was divided into 4 score ranks: 0%‐5% (0), 6% to 25% (1), 26% to 50% (2), 51% to 75% (3) and 76%‐100% (4). The staining intensity was divided into 4 score ranks: negative (0), light brown (1), brown (2) and dark brown (3). The immunoreactivity scores (IRSs) = extent score × intensity score. An IRS of ≤3 was defined as negative, and a score of >3 was defined as positive.

### 2.9 Total Protein extraction and Western blot

The detailed protocol was performed as described previously.^21^ The antibody information was presented in Supplementary Table S2. Each experiment was performed in triplicate.

### 2.10 Immunofluorescence and immunocytochemistry

For Immunofluorescence (IF), the cells were fixed with 4% paraformaldehyde for 20 minutes and permeabilized with 0.2% Triton X‐100 for 10 minutes. After blocking with 5% bovine serum albumin (BSA) for 30 minutes at room temperature, the cells were incubated at 4°C overnight with primary antibodies against E‐cadherin, vimentin and STAT3 (1:100 dilution). The dishes were washed three times with PBS for 10 minutes each and then incubated with Alexa Fluor 594‐conjugated secondary antibodies (1:400 dilution; Invitrogen) for 1 hour at room temperature. The nuclei were stained with DAPI (10 μg/mL) for 10 minutes. The samples were examined via microscopy (Leica Microsystems) to analyse the expression of E‐cadherin and vimentin and the nuclear translocation of STAT3.

For immunocytochemistry (ICC), the cells were fixed with 4% paraformaldehyde for 20 minutes and permeabilized with 0.2% Triton X‐100 for 10 minutes and then incubated with the primary antibodies against E‐cadherin and vimentin. The following protocol is the same as the Immunohistochemistry (IHC) assay.

### 2.11 Luciferase reporter assay

For promoter analyses, a fragment of the SOCS3 5′‐flanking sequence (from −3182 bp to + 425 bp) and other truncated fragments were cloned into the luciferase reporter vector pGL3.0 Basic Vector (Promega) to generate a SOCS3 full promoter reporter construct and the truncated ones. The primers for plasmid constructs were presented in Supplementary Table S1. The detailed protocol was performed as described previously.^21^ Each experiment was performed in triplicate.

### 2.12 Quantitative chromatin immunoprecipitation

Cells were subjected to ChIP using the EZ‐ChIP Kit (Millipore).

The detailed protocol was performed as described previously.^21^ Real‐time PCR was conducted to amplify the regions of DNA fragments by using special primers (Supplementary Table S1). Each experiment was performed in triplicate.

### 2.13 Statistical analysis

The differences among the groups were compared by Student's *t*‐test or one‐way ANOVA. All statistical analyses were performed using the SPSS statistical package (SPSS Inc, Chicago, IL, USA). *P* < .05 was considered statistically significant.

## 3 RESULTS

### 3.1 FXR activation inhibits the proliferation of colon cancer cells in vitro

**Figure 1:**
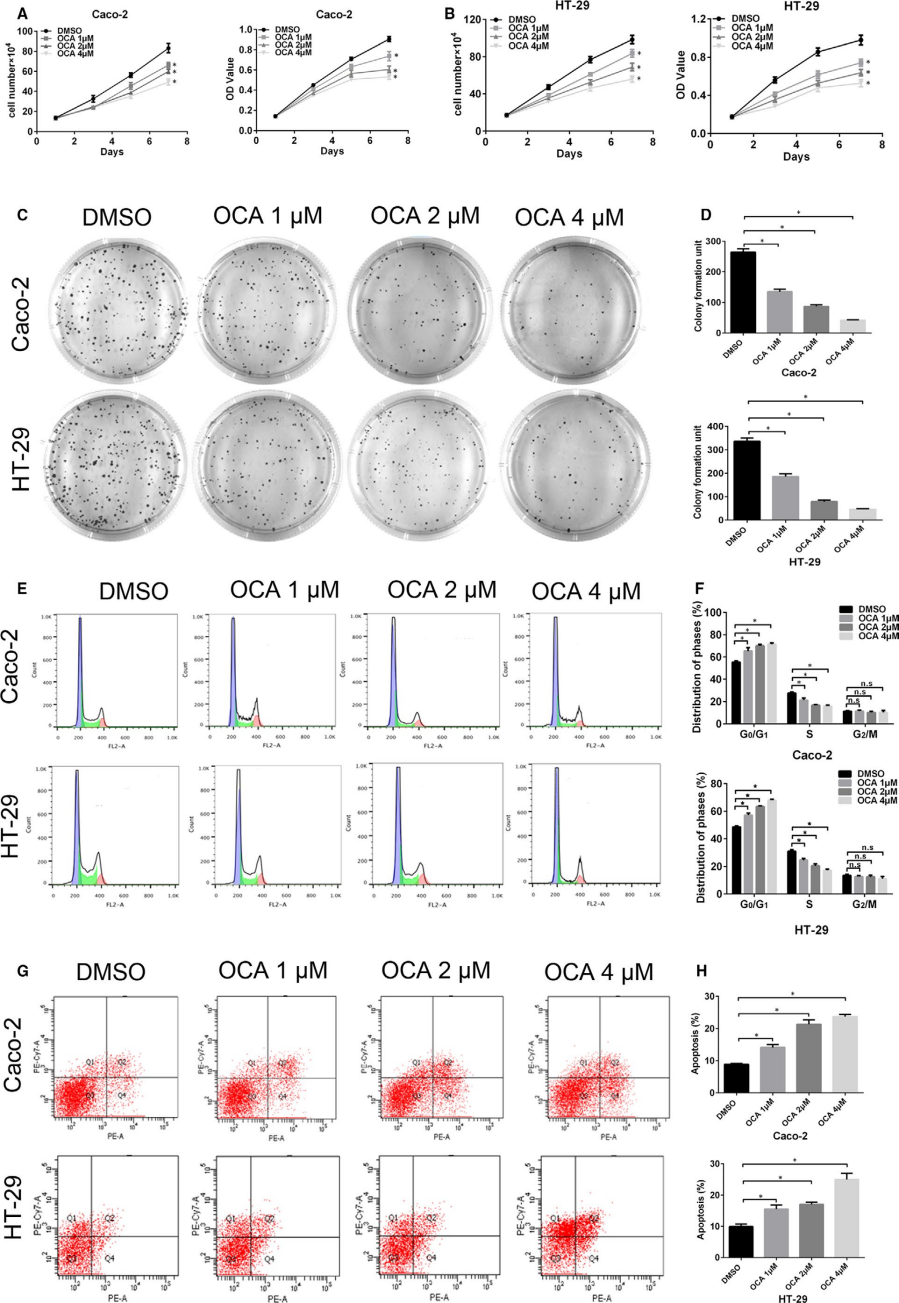
Farnesoid X receptor (FXR) activation inhibits the proliferation of colon cancer cells by inducing cell cycle arrest and apoptosis. A and B, Effect of different doses of obeticholic acid (OCA) on the growth and viability of Caco‐2 (A) and HT‐29 (B) cells detected by cell growth and CCK8 assays. C, Results of colony formation assays for Caco‐2 and HT‐29 cells after OCA exposure for 14 d. D, Quantification of the cell colonies. E, FACS analysis of the cell cycle distribution of Caco‐2 and HT‐29 cells after OCA exposure. F, Comparison of the cell cycle distribution. G, FACS analysis of PE/APC staining for apoptosis in Caco‐2 and HT‐29 cells after OCA exposure. H, Quantification of apoptotic cells. *P < .05

The effect of FXR activation on tumour cell viability and growth was assessed by cell growth curve and CCK8 assays. The results showed that OCA treatment dose dependently inhibited the viability and growth of HT‐29 and Caco‐2 cells in relation to the control (Figure 1A,B). This inhibitory effect was further confirmed by the colony formation assay, in which OCA treatment dose‐dependent reduced the colony formation (Figure 1C,D).

As cell proliferation inhibition was observed after OCA treatment, cell cycle distribution and apoptotic state were assessed by flow cytometry assay. OCA treatment resulted in a dose‐dependent accumulation of cells in the G0/G1 phase with a decrease in cells in the S phase (Figure 1E,F). However, OCA had no significant impact on the accumulation of cells in the G2 phase. Moreover, exposure to OCA dose dependently increased the apoptotic rate of colon cancer cells (Figure 1G,H).

We then examined the impact of OCA treatment on the key proteins associated with G1/S transition and apoptosis. Western blot analysis showed that OCA treatment dose dependently elevated the levels of p21 and active caspase‐3 and reduced the levels of cyclin D1 and c‐Myc (Figure S1A‐D). Altogether, these data suggested that FXR activation retards colon cancer cell proliferation by preventing G1/S transition and inducing apoptosis.

### 3.2 FXR activation retards the tumour growth in nude mice

**Figure 2:**
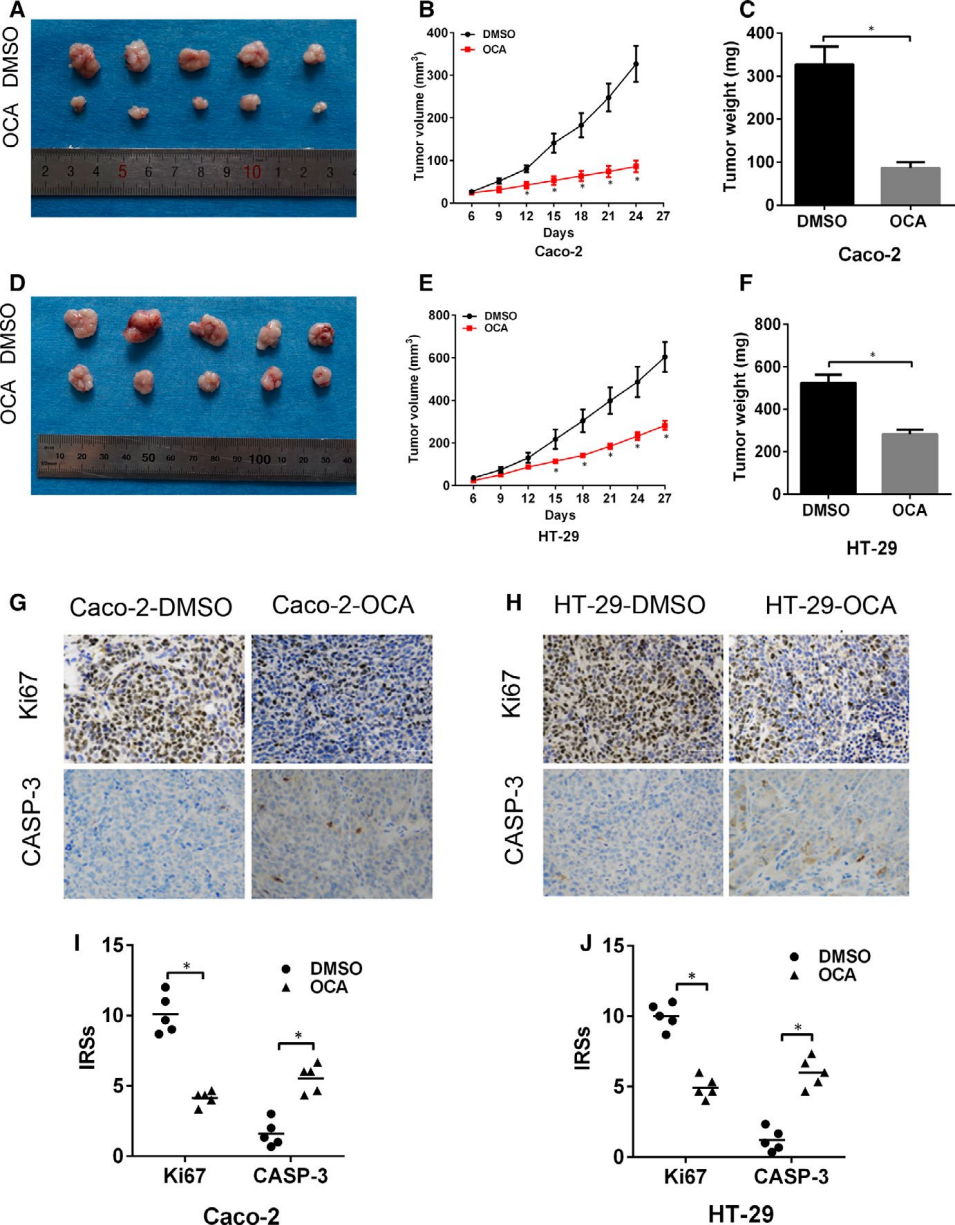
Farnesoid X receptor (FXR) activation retards the tumour growth in nude mice. A, Image of xenograft tumours formed by Caco‐2 cells after obeticholic acid (OCA) treatment. B and C, Tumour growth curves (B) and tumour weights (C) for Caco‐2 cells after OCA treatment. D, Image of xenograft tumours formed by HT‐29 cells after OCA treatment E and F, Tumour growth curves (E) and tumour weights (F) for HT‐29 cells after OCA treatment. G and H, Immunohistochemical staining for Ki67 (G) and active caspase‐3 (H) in tumour xenografts of Caco‐2 (B) cells. I and J, Immunohistochemical staining for Ki67 (I) and active caspase‐3 (J) in tumour xenografts of HT‐29 cells. *P < .05

Next, xenograft mouse model was conducted to assess the effect of FXR activation on tumour growth in vivo. The xenograft tumours in the OCA‐treated group showed a decline in growth rate in relative to the DMSO‐treated group (Figure 2A,B,D,E). Moreover, mean weight of xenograft tumours in the OCA‐treated group is lighter than that in the DMSO‐treated group (Figure 2C,F). Ki67 and active caspase‐3 are well‐known markers evaluating cellular proliferation and apoptosis. Thus, we detected Ki67 and active caspase‐3 expression in xenograft tumours by using IHC staining. The xenograft tumour tissues in the OCA‐treated group displayed weaker Ki67 (Figure 2G,H) and stronger caspase‐3 staining intensity (Figure 2I,J) than those in the DMSO‐treated group. Collectively, our data indicated that FXR activation retards the tumour growth in nude mice.

### 3.3 FXR activation inhibits the invasion and migration of colon cancer cells by arresting EMT

**Figure 3:**
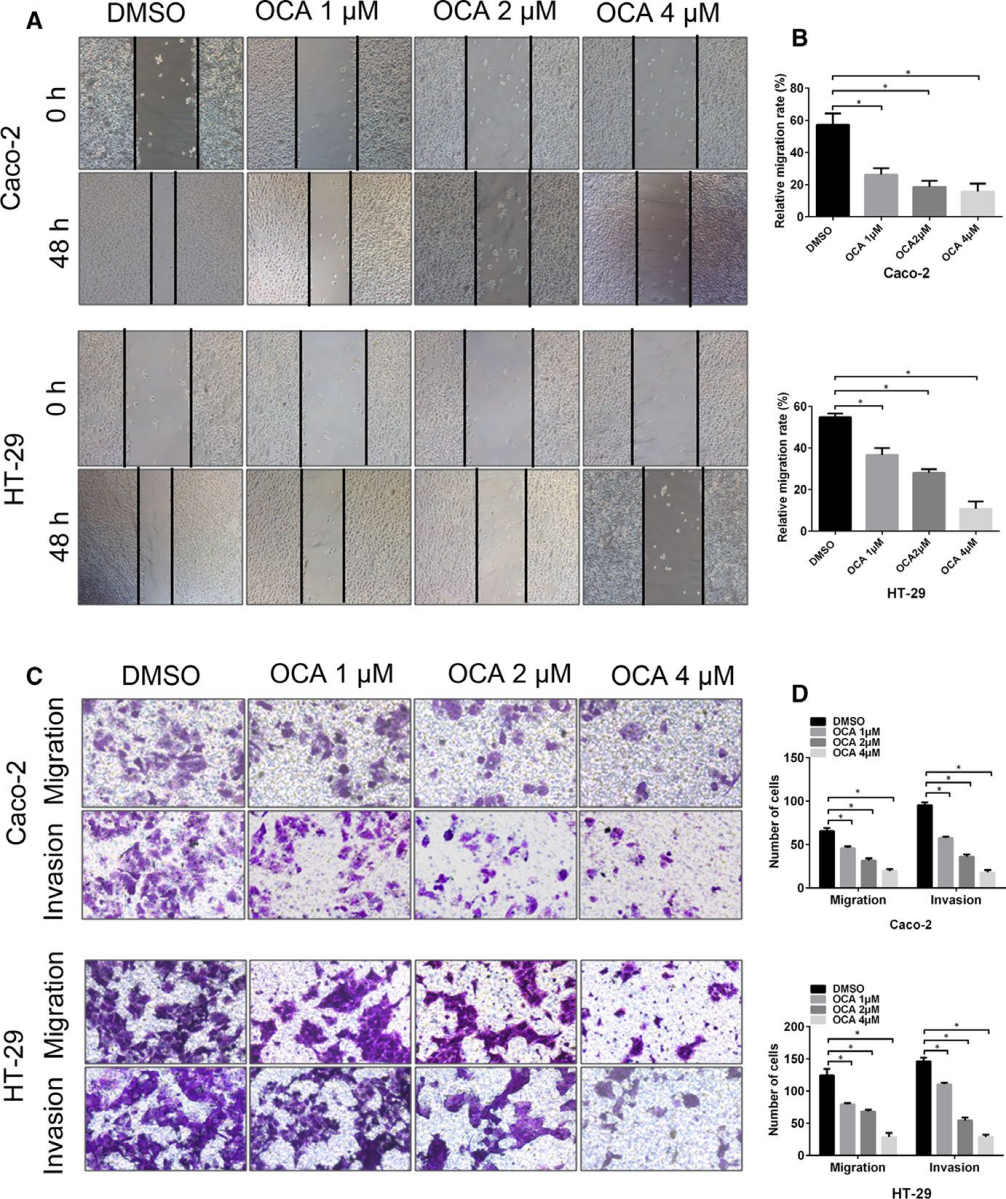
Farnesoid X receptor (FXR) activation inhibits the invasion and migration of colon cancer cells in vitro. A, Wound‐healing assay in Caco‐2 and HT‐29 cells after obeticholic acid (OCA) exposure measured at 48 h. B, The percentage of wound healing in Caco‐2 and HT‐29 cells. C, Invasion and migration assays in Caco‐2 and HT‐29 cells after OCA exposure. D, The number of invasive and migrating cells in Caco‐2 and HT‐29 cells. *P < .05

Considering that tumour metastasis is the leading cause of cancer‐related death in CRC, we thus aimed to evaluate the impact of FXR activation on the invasive and migratory abilities of colon cancer cells. The wound‐healing assay showed that OCA treatment resulted in a decreased rate of wound healing (Figure 3A,B). The Transwell assays showed that the number of invasive and migrating cells in the OCA‐treated group was less than that in the DMSO‐treated group (Figure 3C,D). Epithelial‐mesenchymal transition (EMT) has been linked to the mobility and dissemination of CRC by conferring increased invasiveness and metastatic potential to cells.^23^, ^24^ Our study further assessed the levels of EMT‐related genes by using real‐time PCR and Western blotting analysis. OCA treatment increased the mRNA and protein levels of E‐cadherin and reduced vimentin, N‐cadherin and MMP9 levels (Figure S2A‐F). Furthermore, ICC (Figure S3A,B) and IF analyses (Figure S3C‐F) showed that OCA‐treated cells had a stronger E‐cadherin staining and a weaker vimentin staining than DMSO‐treated cells. Collectively, our data indicated that FXR activation inhibits the invasive and migratory ability of colon cancer cells by arresting EMT.

### 3.4 FXR activation suppresses JAK2/STAT3 pathway by up‐regulating SOCS3 expression in colon cancer cells

**Figure 4:**
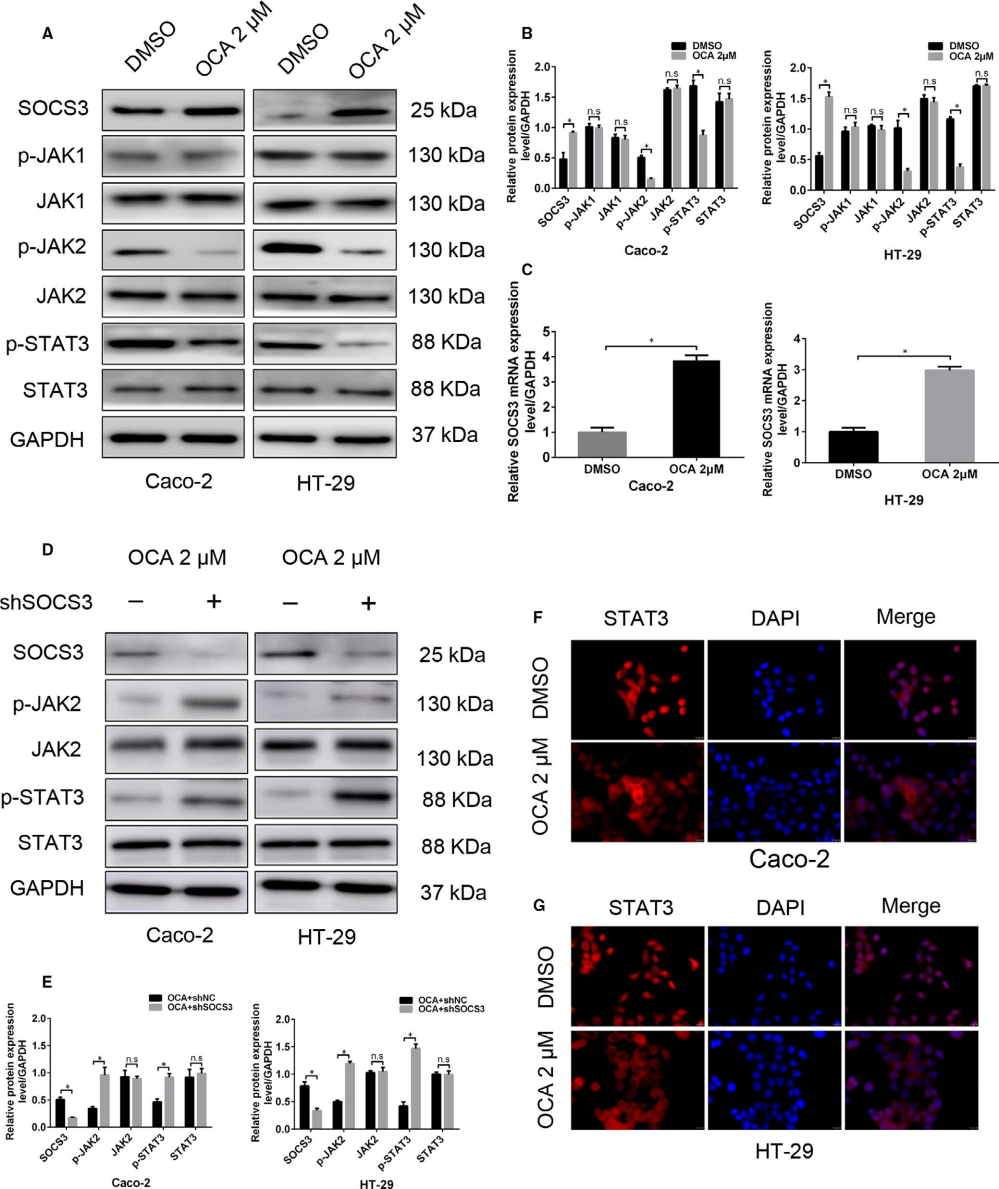
Farnesoid X receptor (FXR) activation suppresses JAK2/STAT3 pathway by up‐regulating SOCS3 expression in colon cancer cells. A, Western blot bands of the critical proteins of JAK/STAT pathway in Caco‐2 and HT‐29 cells after obeticholic acid (OCA) exposure. B, Quantitative analysis of the critical proteins of JAK/STAT pathway in Caco‐2 and HT‐29 cells. C, Real‐time PCR of SOCS3 levels in Caco‐2 and HT‐29 cells after OCA exposure. D, Western blot bands of the critical proteins of JAK/STAT pathway in OCA‐treated Caco‐2 and HT‐29 cells upon SOCS3 knockdown. E, Quantitative analysis of the critical proteins of JAK/STAT pathway in Caco‐2 and HT‐29 cells. F and G, Immunofluorescence staining of STAT3 in Caco‐2 (F) and HT‐29 (G) after OCA exposure. *P < .05

The Janus tyrosine kinases/signal transducer and activator of transcription (JAK/STAT) pathway, which is negatively regulated by the SOCS family, plays a crucial role in cellular responses by regulating survival, proliferation, invasion and differentiation.^25^, ^26^ Our study attempted to detect the impact of FXR activation on the activity of JAK/STAT signalling. OCA treatment inhibited the protein expression of p‐STAT3 and p‐JAK2 (Figure 4A,B). However, OCA treatment had no significant impact on phosphorylated JAK1 expression (Figure 4A,B). Furthermore, we found that OCA treatment resulted in an up‐regulation of SOCS3 expression at both mRNA and protein levels (Figure 4A,B). To further validate the involvement of SOCS3 in OCA‐mediated inhibition of JAK/STAT signalling, we knocked down SOCS3 in colon cancer cells. Depleting SOCS3 expression restored the activity of JAK/STAT signalling in OCA‐treated cells (Figure 4D,E).

Phosphorylated STATs dimerize and translate into the nucleus to regulate target gene transcription.^27^ Thus, we attempted to assess the localization of STAT3 protein by using IF staining. Exposure to OCA resulted in increased STAT3 protein accumulation in the cell cytoplasm (Figure 4F,G). Taken together, these results suggest that FXR activation might suppress JAK2/STAT3 pathway in colon cancer cells by up‐regulating SOCS3 expression.

**Figure 5:**
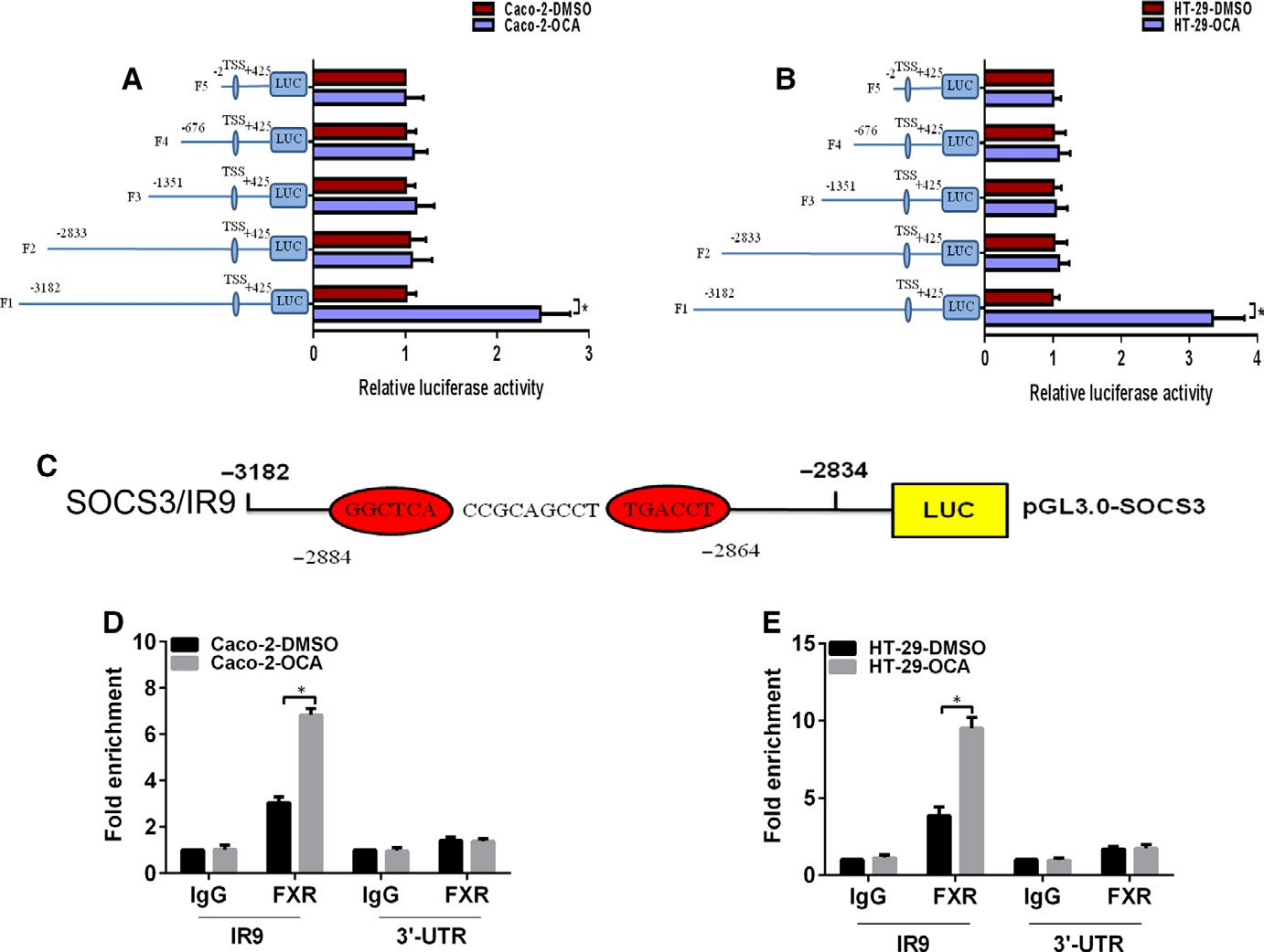
Farnesoid X receptor (FXR) transactivates the expression of SOCS3 by binding to the FXRE/IR9 in the SOCS3 promoter in colon cancer cells. A and B, The activities of the full SOCS3 promoter reporter construct and the truncated ones in Caco‐2 (A) and HT‐29 (B) cells after obeticholic acid (OCA) exposure using the dual‐luciferase assay. C, Schematic representation of a putative FXRE in the human SOCS3 promoter region (SOCS3/IR9). Enrichment level of FXR binding to the putative FXRE in the SOCS3 promoter region in Caco‐2 (D) and HT‐29 (E) cells determined by the qChIP assay. *P < .05

It has been demonstrated that FXR usually modulates target genes transcription by specifically binding to FXR response element (FXRE).^28^ We employed the dual‐luciferase reporter assay to assess the impact of FXR activation on SOCS3 transcription. FXR‐activating cells transfected with the full‐length SOCS3 promoter displayed a high luciferase intensity in relative to the control cells (Figure 5A,B). However, OCA treatment did not result in the change in the luciferase intensity of the other truncated fragments compared with the control, suggesting that FXR activation could transcriptionally activate SOCS3 expression through binding to the −3182 bp to −2834 bp region of the SOCS3 promoter in colon cancer cells.

Next, we attempted to search for the putative binding site of FXR in the SOCS3 promoter by using JASPAR database. A potential FXRE/IR9 (an inverted repeat spaced by nine nucleotides, −2884 bp to −2864 bp) was identified in the −3182 bp to −2834 bp region of the SOCS3 promoter (Figure 5C). We conducted the Quantitative chromatin immunoprecipitation (qChIP) assay to determine whether OCA treatment facilitates the binding of FXR to the FXRE/IR9 of the SOCS3 promoter. As expected, an enrichment of FXR binding to the FXRE/IR9 was observed in OCA‐treated cells in relative to the control cells (Figure 5D,E). All of these results indicate that FXR activation inhibits JAK2/STAT3 pathway by regulating SOCS3 transcription in colon cancer cells.

### 3.5 Activation of JAK2/STAT3 signalling rescues the tumour‐suppressive effect of FXR activation

**Figure 6:**
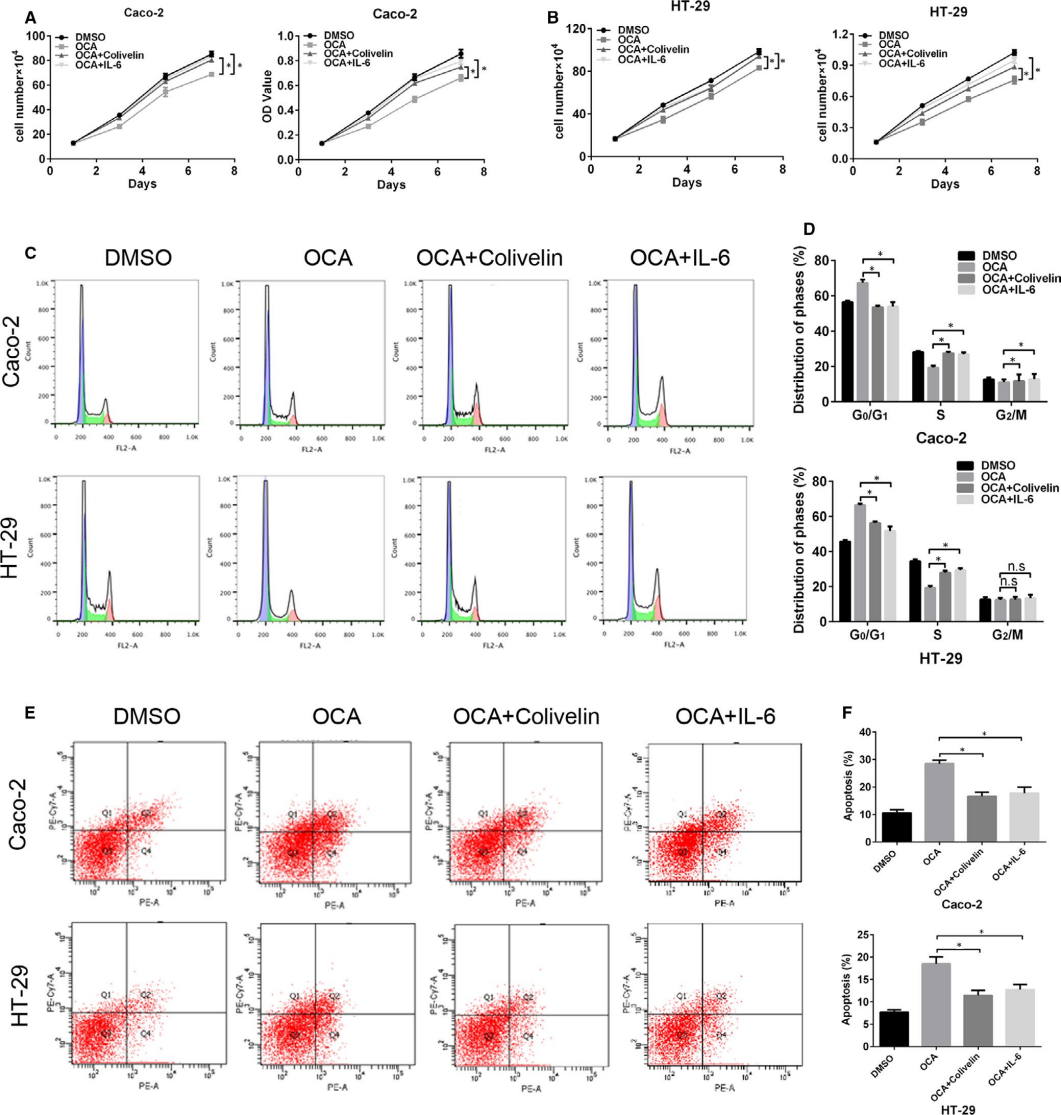
Activation of the JAK2/STAT3 pathway rescues the inhibitory effect of Farnesoid X receptor (FXR) activation on the proliferation of colon cancer cells. A and B, The effect of colivelin and IL‐6 on the growth and viability of FXR‐activated Caco‐2 (A) and HT‐29 (B) cells detected by cell growth and CCK8 assays. C, FACS analysis of the cell cycle distribution of FXR‐activated Caco‐2 and HT‐29 cells after colivelin and IL‐6 exposure. D, Comparison of the cell cycle distribution. E, FACS analysis of PE/APC staining for apoptosis in FXR‐activated Caco‐2 and HT‐29 cells after colivelin and IL‐6 exposure. F, Quantification of apoptotic cells. *P < .05

**Figure 7:**
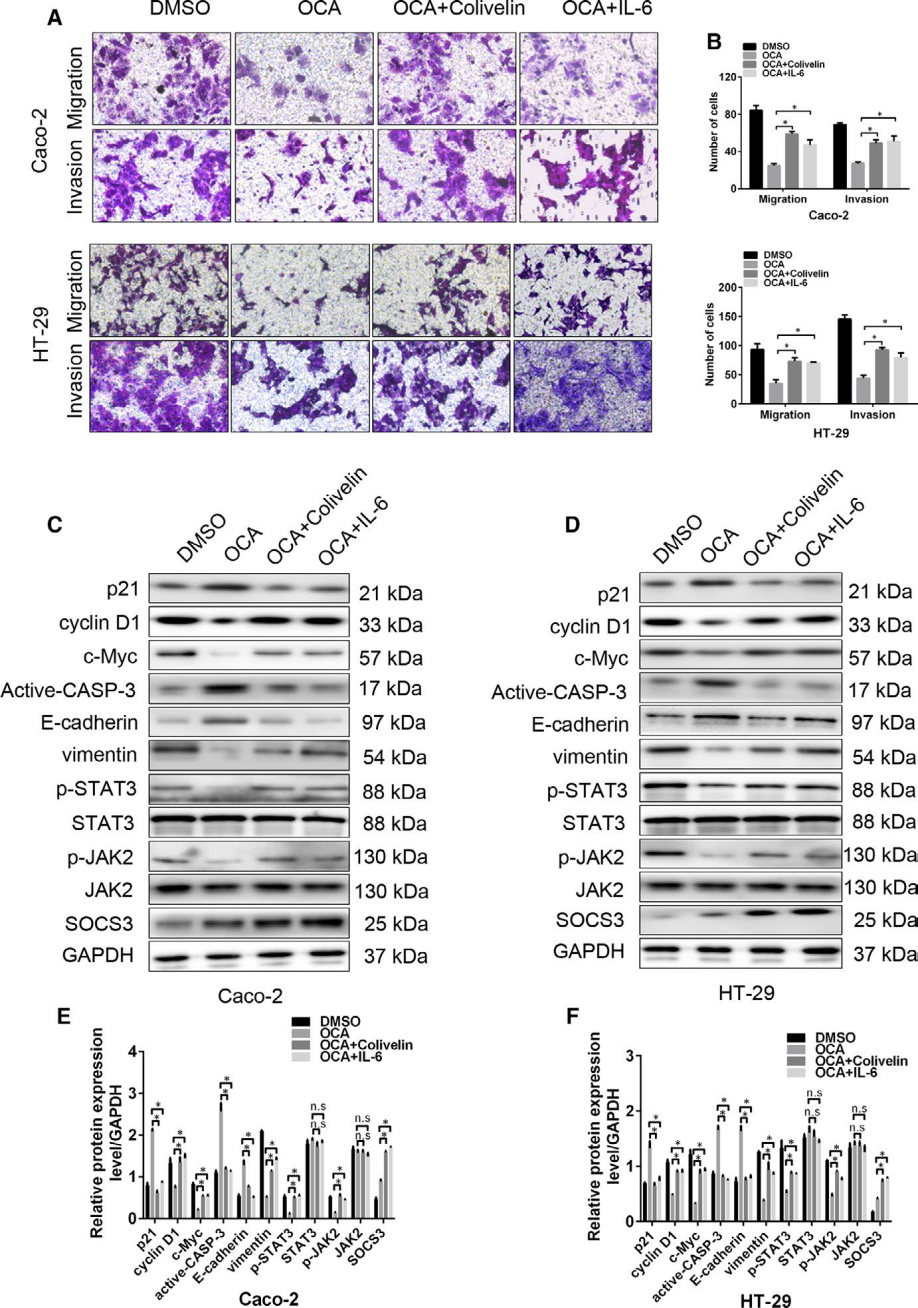
Activation of the JAK2/STAT3 pathway rescues the inhibitory effect of Farnesoid X receptor (FXR) activation on the growth‐, apoptosis‐ and EMT‐related proteins of colon cancer cells. A, The effect of colivelin and IL‐6 on the invasion of FXR‐activated Caco‐2 and HT‐29 detected by invasion and migration assays. B, The number of invasive and migrating cells in Caco‐2 and HT‐29 cells. C and D, Western blot bands of the growth‐, apoptosis‐ and EMT‐related proteins in FXR‐activated Caco‐2 (C) and HT‐29 (D) cells after colivelin and IL‐6 exposure. E and F, Quantitative analysis of the growth‐, apoptosis‐ and EMT‐related proteins in Caco‐2 (E) and HT‐29 (F) cells. *P < .05

In order to determine that JAK2/STAT3 signalling is indispensable for the tumour‐suppressive effect of FXR activation on colon cancer cells, colivelin and IL‐6, the agonists of JAK/STAT3 pathway,^29^, ^30^ were adopted to treat FXR‐activated cells. IL‐6 is a classic STAT3 activator, while colivelin, a potent humanin (HN) derivative, has recently been proved to activate JAK2/STAT3 signalling via binding to cell surface receptors involving CNTFRα, WSX‐1 and gp130.^30^ In the current study, colivelin and IL‐6 rescued the proliferative and invasive abilities inhibited by FXR activation (Figure 6A‐F, Figure 7A,B). Consistent with the observations above, colivelin and IL‐6 up‐regulated the protein levels of p‐STAT3, p‐JAK2, vimentin, c‐Myc and cyclin D1 but reduced the level of p21, active CASP‐3 and E‐cadherin (Figure 7C‐F). Taken together, these results demonstrate that JAK2/STAT3 signalling participates in OCA‐mediated tumour inhibition.

## 4 DISCUSSION

Recently, due to the limited effectiveness of conventional therapeutic strategies, there has been a strong interest in the field of novel antitumour drugs. These novel drugs characterized by the good markers available are the major goals of molecular oncology. The tumour‐suppressive role of FXR in colorectal tumorigenesis has inspired us to restore FXR activity as a novel therapeutical strategy. Our study indicated that FXR activation by OCA inhibited the proliferative potential of colon cancer cells by arresting G1/S transition and inducing apoptosis. In support of these data, treatment with OCA significantly altered the levels of growth‐ and apoptosis‐related proteins. FXR agonist GW4064 has been reported to trigger apoptosis and retard tumour growth by activating its target SHP.^31^ Moreover, restoring FXR expression inhibited cyclin D1 and c‐Myc levels by repressing the Wnt/β‐catenin signalling.^32^

Epithelial‐mesenchymal transition is a biological process that involves the morphological transformation of epithelial cells, with the cells acquiring mesenchymal‐like and motile phenotypes.^33^ OCA treatment impaired the invasive and migratory potential of colon cancer cells by arresting EMT. Inconsistent with the result of our work, Kainuma M et al reported that treatment of HCC with OCA treatment promoted EMT phenotypes,^34^ although FXR may act as a tumour suppressor at the early stage of HCC through the regulation of hepatic inflammatory responses.^20^ Collectively, our study for the first time reported the tumour‐suppressive effect of OCA on CRC.

The highly malignant behaviour of colon cancer cells is closely associated with the activity of JAK/STAT pathway.^35^ Our data indicated that OCA treatment repressed the activation of JAK2/STAT3 pathway in colon cancer cells, which was further confirmed by IF staining, showing increased levels of STAT3 protein located in the cell cytoplasm. In addition, restoration of JAK2/STAT3 signalling by colivelin and IL‐6 abolished OCA‐mediated proliferation and invasion inhibition. Intriguingly, the concentration of colivelin used in CRC and oesophageal cancer^36^ in vitro is much higher than that used in neurodegenerative disease, which may reflect that the regulatory mechanism antagonizing JAK/STAT signalling exists in different disease models. FXR deficiency in HCC results in a high activity of STAT3.^37^ Following this activation step, phosphorylated STATs dimerize and translate into the nucleus to regulate the transcription of target genes related to CRC progression.

SOCS3, an important member of the SOCS family, negatively regulates JAK/STAT pathway.^38^ SOCS3 expression is reduced in CRC tissues due to promoter methylation.^39^ Lack of SOCS3 contributes to accelerative intestinal crypt growth and facilitates tumour growth.^40^ In the current study, OCA treatment increased the mRNA and protein levels of SOCS3. Similarly, restoration of SOCS3 by FXR agonist GW4064 is observed in hepatocellular inflammation and HCC,^41^, ^42^ indicating that FXR‐SOCS3 axis may serve as a new potential target for the prevention and treatment of HCC. Furthermore, our study revealed that OCA treatment could promote SOCS3 transcription by binding to the −3182 bp to −2834 bp of the SOCS3 promoter, as determined by the dual‐luciferase reporter assay. Nuclear hormone receptors regulate gene transcription by recognizing the motif consisting of repeats with the core element AGGTCA aparted by one or more nucleotides.^43^ We predicted a potential FXRE/IR9 (−2884 bp to −2864 bp) in the −3182 bp to −2834 bp of the SOCS3 promoter by using the JASPAR database. The qChIP assay demonstrated that FXR specifically and directly binds to the FXRE/IR9 of SOCS3 promoter. Some pharmacological compounds have been shown to repress STAT3 activity by restoring SOCS3 expression.^44^, ^45^, ^46^ Manipulation of SOCS3 expression via the regulatory role of FXR might be expected to be a promising therapeutic option for cancer chemotherapy. Intriguingly, the activation of FXR by GW4064 decreased the expression of HER2, the upstream of JAK/STAT3 pathway.^47^ Besides JAK/STAT signalling, OCA might exert tumour suppressor role by directly regulating FXR target genes SHP^31^ and MMP7^48^ or suppressing Wnt/β‐catenin^49^ and EGFR/ERK signalling.^50^ Hence, OCA exerts its tumour suppressor functions via multiple molecular mechanisms.

In the present study, the FXR agonist OCA inhibited colon cancer cell proliferation and invasion by repressing JAK2/STAT3 pathway via regulating SOCS3 transcription. Our study provided the first evidence that FXR activation by OCA may represent a potential novel therapeutic strategy for CRC treatment.

## CONFLICT OF INTEREST

All authors declare no conflict of interest.

## AUTHOR CONTRIBUTIONS

**Shan Li:** Project administration (equal); Writing‐original draft (lead); Writing‐review & editing (equal). **Zhengshui Xu:** Project administration (equal); Software (lead); Writing‐review & editing (equal). **Jing Guo:** Methodology (equal); Project administration (equal); Software (lead); Validation (lead). **Jianbao Zheng:** Funding acquisition (equal); Resources (equal); Writing‐review & editing (equal). **Xuejun Sun:** Conceptualization (equal); Funding acquisition (lead); Supervision (lead). **Junhui Yu:** Conceptualization (lead); Methodology (lead); Project administration (equal); Supervision (equal); Writing‐original draft (equal); Writing‐review & editing (lead).

## Supporting information

Supplementary Material

Figure S1

Figure S2

Figure S3

## Data Availability

The data used to support the findings of this study are included within the article.
