# Supplementary material for: Farnesoid X receptor activation induces antitumour activity in colorectal cancer by suppressing JAK2/STAT3 signalling via transactivation of SOCS3 gene
Source: J Cell Mol Med. 2020 Nov 9;24(24):14549–60. doi: 10.1111/jcmm.16083 (PMC7754034; doi:10.1111/jcmm.16083)
Supplement: Supplementary file 1 — Supplementary Material [file JCMM-24-14549-s001.docx]

Table S1 Primer sequence

| Gene | Sequence |
| --- | --- |
| RT-PCR |  |
| SOCS3 | F: 5’-ACCAGCGCCACTTCTTCACG-3’ |
|  | R: 5’-GTGGAGCATCATACTGATCC-3’ |
| Luciferase Assays |  |
| F1 (-3182-+425) | F: 5’-ATGGCGCGATCTCGGCTCAC-3’ |
| F2 (-2833-+425) | F 5’-CTCCCAAGCAGCTGGGACT-3’ |
| F3 (-1351-+425) | F 5’-GCCTCTGCCAGAAATCAGCCT-3’ |
| F4 (-676-+425) | F 5’-GGAGACTTCGATTCGGGACCAG-3’ |
| F5 (-2-+425) | F 5’-CCATGGTCACCCACAGCAAGT-3’ |
|  | R 5’-TCAGTAGGTGGCGAGGGGAAG-3’ |
| ChIP-qPCR  IR9/FXRE | F 5’-TAACAGGGTCTCACTCTGTTGCC-3’ |
|  | R 5’-TAGCCAGGAGTGGTGGCCT-3’ |
| 3’-UTR | F 5’-AAGCCCAGACATGGGGTCCT-3’ |
|  | R 5’-GGTGATCCTGTCCGCTGCTC-3’ |

Table S2 Antibodies

| Antibodies | Source | | Identifier |
| --- | --- | --- | --- |
| GAPDH | | Santa Cruz | Cat#sc-47724 |
| FXR | | Santa Cruz | Cat#sc-25309 |
| N-cadherin | | Abcam | Cat#ab18203 |
| vimentin | | Abcam | Cat#ab92547 |
| MMP9 | | Abcam | Cat#ab76003 |
| caspase-3 | | Abcam | Cat#ab179517 |
| JAK1 | | Abcam | Cat#ab133666 |
| p-JAK1(Tyr1022/1023) | | Abcam | Cat#ab138005 |
| JAK2 | | Abcam | Cat#ab108596 |
| p-JAK2(Tyr1007/1008) | | Abcam | Cat#ab32101 |
| STAT3 | | Abcam | Cat#ab68153 |
| p-STAT3(Tyr705) | | Abcam | Cat#ab76315 |
| SOCS3 | | Cell Signaling Technology | Cat#52113 |
| E-cadherin | | Cell Signaling Technology | Cat#14472 |
| cyclin D1 | | Cell Signaling Technology | Cat#55506 |
| c-Myc | | Cell Signaling Technology | Cat#18583 |
| p21 | | Cell Signaling Technology | Cat#2947 |

**Supplementary Fig. 1 FXR activation affects the protein levels of growth- and apoptosis-related genes.** a, c. Western blot bands of the growth- and apoptosis-related proteins in Caco-2 (a) and HT-29 (c) cells after OCA exposure. b, d Quantitative analysis of the growth- and apoptosis-related proteins in Caco-2 (b) and HT-29 (d) cells. * P<0.05.

**Supplementary Fig. 2 FXR activation inhibits EMT in colon cancer cells.** a, d. Real-time PCR in Caco-2 (a) and HT-29 (d) cells after OCA exposure. b, e. Western blot bands of the EMT-related proteins in Caco-2 (b) and HT-29 (e) cells after OCA exposure. c, f. Quantitative analysis of EMT-related proteins in Caco-2 (c) and HT-29 (f) cells. * P<0.05

**Supplementary Fig. 3 FXR activation increases the expression of E-cadherin but reduces the expression of vimentin.** a, b. Immunocytochemistry staining of E-cadherin and vimentin in Caco-2 (a) and HT-29 (b) cells after OCA exposure. c, d. Immunofluorescence staining of E-cadherin (c) and vimentin (d) in Caco-2 cells after OCA exposure. e, f. Immunofluorescence staining of E-cadherin (e) and vimentin (f) in HT-29 cells after OCA exposure.
