# Supplementary figures and images for: Farnesoid X receptor activation induces antitumour activity in colorectal cancer by suppressing JAK2/STAT3 signalling via transactivation of SOCS3 gene
Source: J Cell Mol Med. 2020 Nov 9;24(24):14549–60. doi: 10.1111/jcmm.16083 (PMC7754034; doi:10.1111/jcmm.16083)

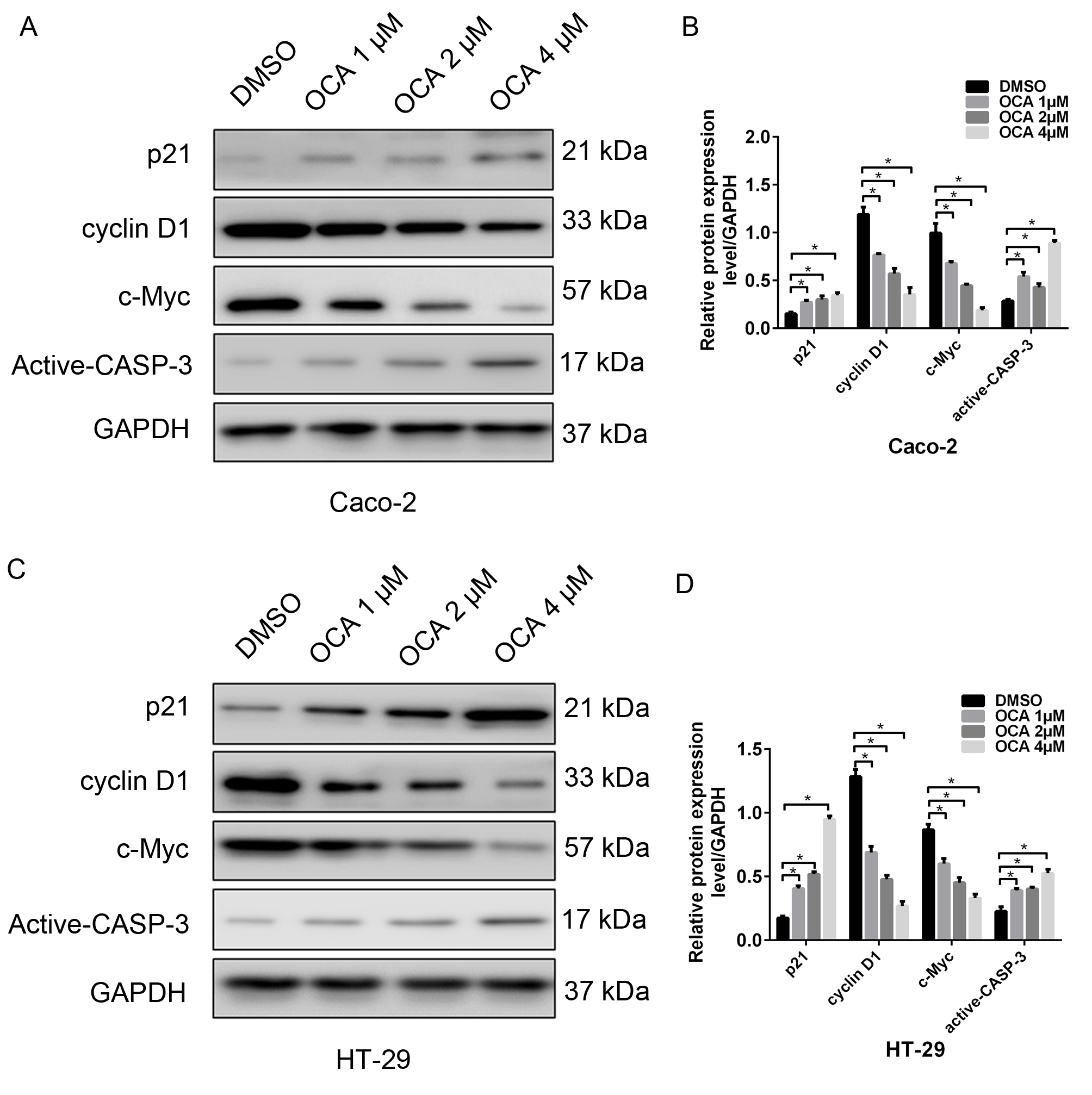

Supplement: Supplementary file 2 — Figure S1 [file JCMM-24-14549-s002.tif]

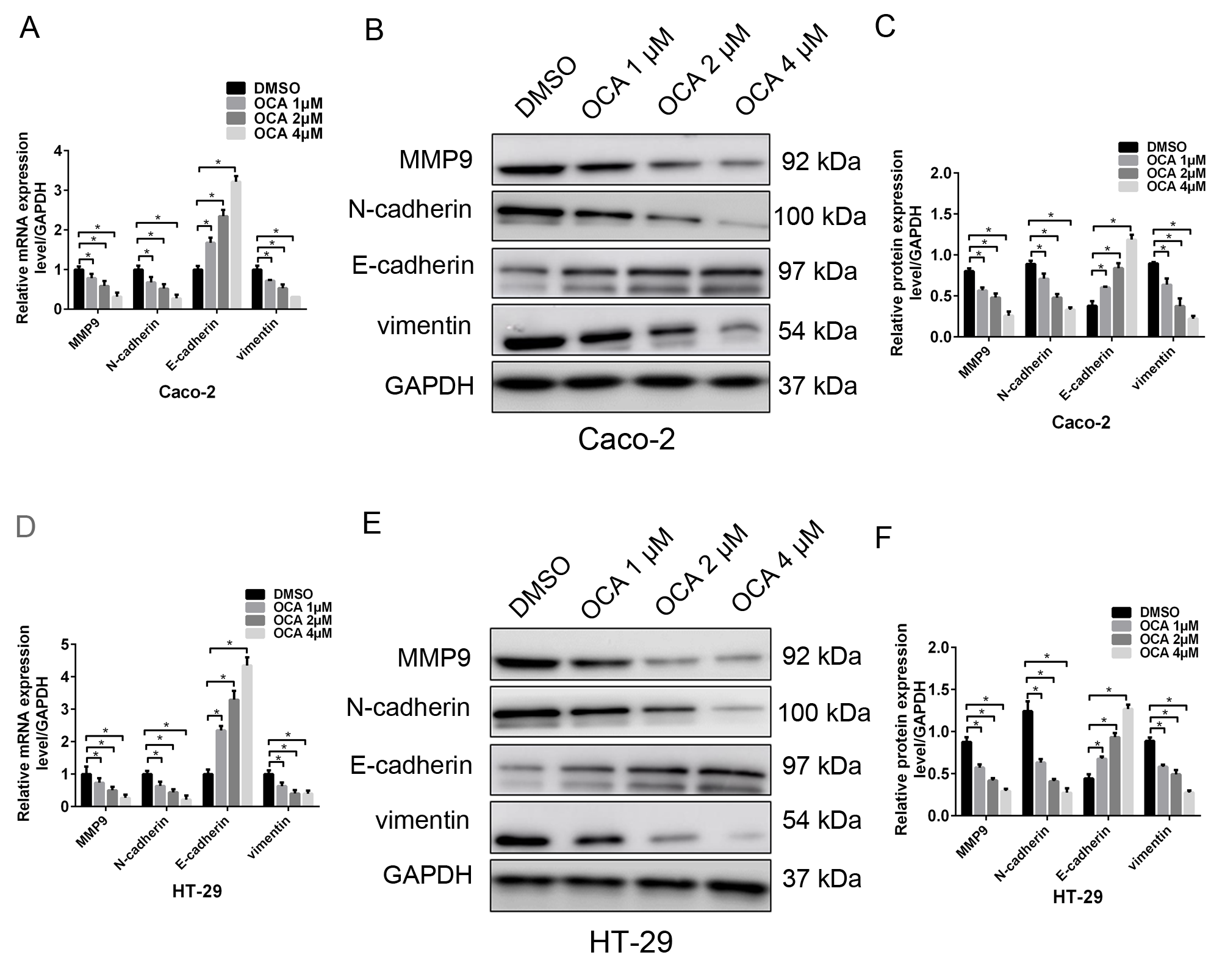

Supplement: Supplementary file 3 — Figure S2 [file JCMM-24-14549-s003.tif]

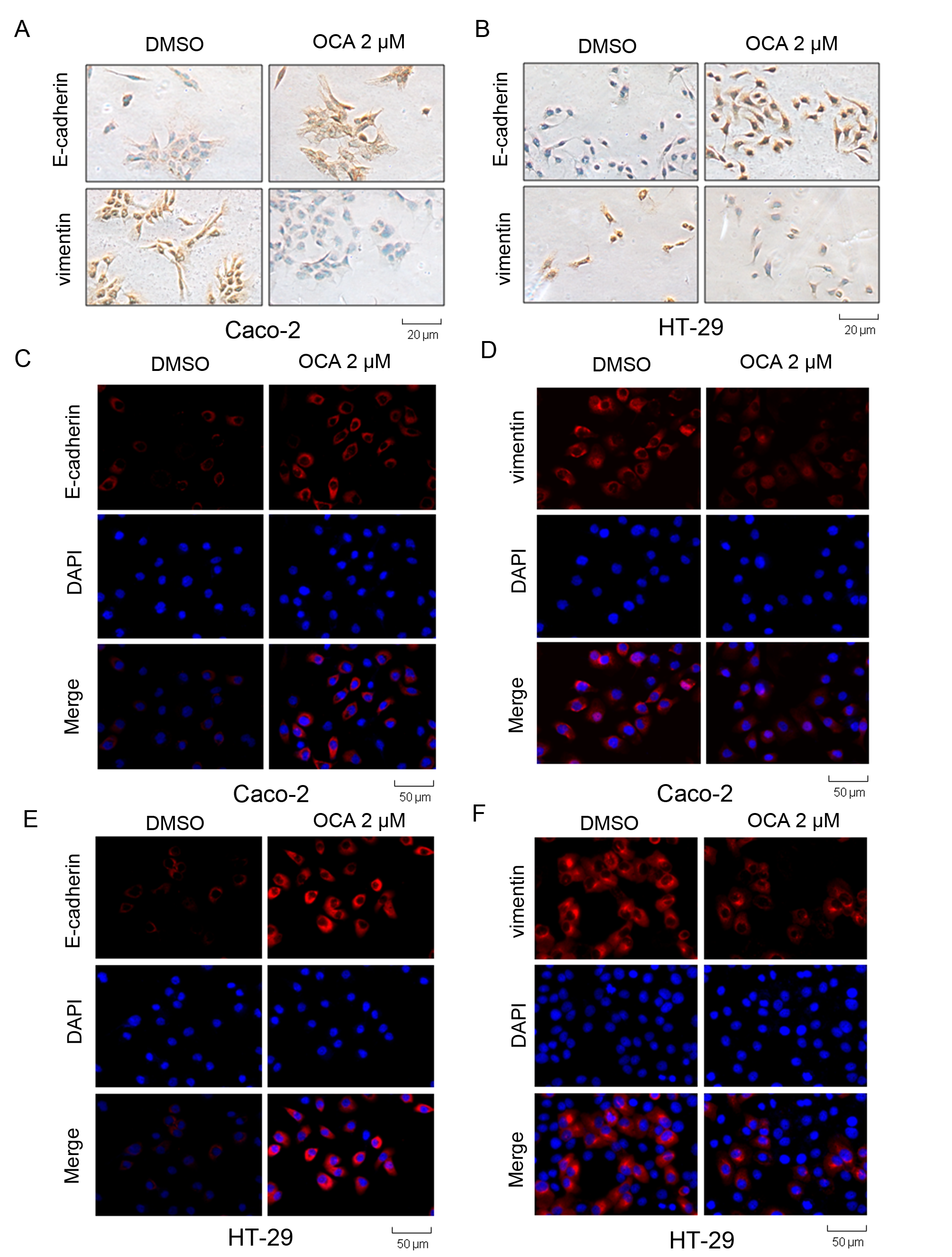

Supplement: Supplementary file 4 — Figure S3 [file JCMM-24-14549-s004.tif]
